# The clinical spectrum of HTLV-1 infection

**DOI:** 10.1186/1742-4690-12-S1-O19

**Published:** 2015-08-28

**Authors:** Davi Tanajura, Abraão Neto, Isadora Siqueira, Valéria Gusmão, Paulo Oliveira, Silvana Giozza, Neviton Castro, Natalia Carvalho, Silvane Santos, Edgar M Carvalho

**Affiliations:** 1 Serviço de Imunologia - Hospital Universitário Professor Edgard Santos, Salvador, Bahia, Brazil; 2 Centro de Pesquisa Gonçalo Muniz – FIOCRUZ, Salvador, Bahia, Brazil; 3 Faculdade de Medicina da Bahia – UFBA, Salvador, Bahia, Brazil

## 

The Human T lymphotropic virus type 1 (HTLV-1) infection is neglected mainly due to the concept that it is associated with a low morbidity. However, a few reports have shown that a large percentage of HTLV-1 infected subjects have signs and symptoms of a variety of diseases. Nevertheless the relationship between the cause and effect needs to be proven. The HTLV-1 associated myelopathy (HAM/TSP) and adult T-cell leukemia (ATL), the main disease associated to HTLV-1, are characterized by high proviral load and lymphocyte activation. Moreover the exaggerated inflammatory response and proviral load are markers of diseases associated with HTLV-1. In this study we compare the frequency of sicca syndrome, chronic periodontal disease (CPD), HTLV-1 associated over reactive bladder (HTLV-1 OAB), artropathy and erectile dysfunction (ED) in HTLV-1infected subjects who did not have definitive HAM/TSP with that observed in seronegative controls. The proviral load was measured by real time PCR and production of interferon–γ and TNF in supernatants of lymphocyte culture by ELISA. Of the 180 individuals participants of the study, 106 (50.8%) were female, 56 (31.1%) had sicca syndrome, 37 (20.5%) had CPD, 32 (17.7%) had HTLV-1 OAB, 29 (16.6%) had HTLV-1 associated arthropathy. Moreover of the 74 males 31 (41.8%) had ED. All these manifestations were higher (P<0.001) in HTLV-1 infected subjects than in controls. Proviral load in sicca syndrome, CPD, HTLV-1 OAB and ED patients were higher (P < 0.05) than in 78 (43,3%) HTLV-1 carriers. Patients with OAB, CPD and sicca syndrome had higher TNF and IFN-γ than HTLV1 carriers. The majority of the patients with diseases associated to HTLV1 had at least two of the above diseases. Overall 56,7% of HTLV-1 infected subjects without definitive HAM/TSP or ATL had diseases associated with HTLV1 infection indicating that HTLV-1 is associated with high morbidity.

